# Inhibition of Enzymatic Acetylation-Mediated Resistance to Plazomicin by Silver Ions

**DOI:** 10.3390/ph16020236

**Published:** 2023-02-03

**Authors:** David Ngo, Angel J. Magaña, Tung Tran, Jan Sklenicka, Kimberly Phan, Brian Eykholt, Verónica Jimenez, María S. Ramirez, Marcelo E. Tolmasky

**Affiliations:** Center for Applied Biotechnology Studies, Department of Biological Science, California State University Fullerton, Fullerton, CA 92831, USA

**Keywords:** AAC(2′)-Ia, aminoglycoside 2′-*N*-acetyltransferase type Ia, aminoglycoside, multidrug resistance, metal ions, plazomicin, adjuvant

## Abstract

Plazomicin is a recent U.S. Food and Drug Administration (FDA)-approved semisynthetic aminoglycoside. Its structure consists of a sisomicin scaffold modified by adding a 2(S)-hydroxy aminobutyryl group at the N1 position and a hydroxyethyl substituent at the 6′ position. These substitutions produced a molecule refractory to most aminoglycoside-modifying enzymes. The main enzyme within this group that recognizes plazomicin as substrate is the aminoglycoside 2′-*N*-acetyltransferase type Ia [AAC(2′)-Ia], which reduces the antibiotic’s potency. Designing formulations that combine an antimicrobial with an inhibitor of resistance is a recognized strategy to extend the useful life of existing antibiotics. We have recently found that several metal ions inhibit the enzymatic inactivation of numerous aminoglycosides mediated by the aminoglycoside 6′-*N*-acetyltransferase type Ib [AAC(6′)-Ib]. In particular, Ag^+^, which also enhances the effect of aminoglycosides by other mechanisms, is very effective in interfering with AAC(6′)-Ib-mediated resistance to amikacin. Here we report that silver acetate is a potent inhibitor of AAC(2′)-Ia-mediated acetylation of plazomicin in vitro, and it reduces resistance levels of *Escherichia coli* carrying *aac(2′)-Ia*. The resistance reversion assays produced equivalent results when the structural gene was expressed under the control of the natural or the *bla*_TEM-1_ promoters. The antibiotic effect of plazomicin in combination with silver was bactericidal, and the mix did not show significant toxicity to human embryonic kidney 293 (HEK293) cells.

## 1. Introduction

**Figure 1:**
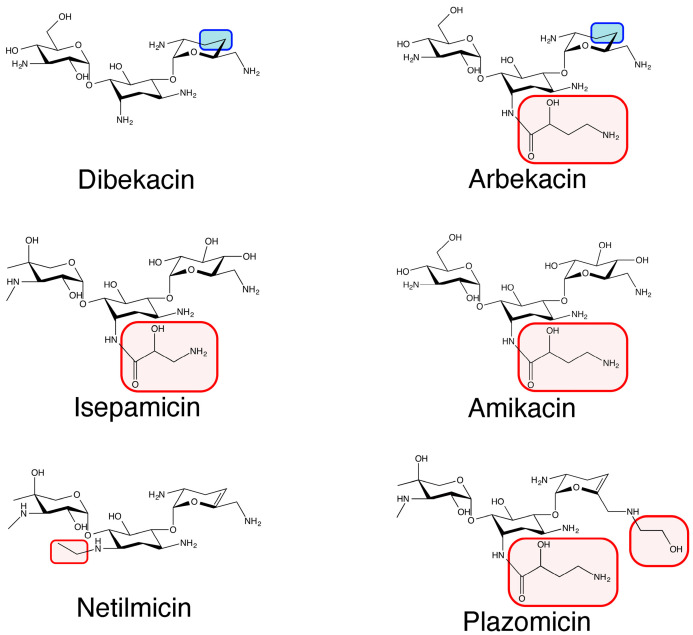
Chemical structures of representative semisynthetic aminoglycosides. Dibekacin is a 3′,4′-dideoxy derivative of kanamycin B [19]. Arbekacin is a derivative of dibekacin obtained by addition of (S)-4-amino-2-hydroxybutyric acid into the amino group at the C-1 position [44] after observing the effect this modification had when carried out on kanamycin B to generate amikacin [21]. Isepamicin is a derivative of gentamicin with a modification like that in amikacin, and the properties of both antibiotics are similar [45]. Netilmicin is a derivative of sisomicin obtained by addition of an ethyl group into the amino group at the C-1 position [20]. Plazomicin is the newest semisynthetic aminoglycoside, generated by modifying sisomicin with the addition of a 2(S)-hydroxy aminobutyryl group at the N1 position and a hydroxyethyl substituent at the 6′ position [26,27]. Red boxes show chemical groups added to the molecule to generate the semisynthetic derivative. Blue boxes show the locations where hydroxyl groups were removed to generate the semisynthetic derivative.

Nosocomial- and community-acquired bacterial pathogens are becoming resistant to most, or even all, available antibiotics, and some are becoming virtually untreatable [1]. Many clinical isolates belonging to the Enterobacterales possess genes coding for aminoglycoside-modifying enzymes, extended-spectrum β-lactamases, and carbapenemases [2,3]. Colistin remains an option for treating life-threatening multidrug-resistant infections caused by some of these bacteria [4]. However, resistant variants have already been found in several geographical regions and may soon become prevalent [5,6]. Other novel options include cefiderocol and new β-lactams/β-lactamase inhibitors [7,8,9]. Aminoglycosides are excellent tools for treating infections caused by Gram-negative and Gram-positive bacteria. Unfortunately, the rise and dissemination of aminoglycoside-modifying enzymes, the major mechanism of resistance to this class of antibiotics in the clinical setting, have reduced their effectiveness [10,11,12]. Therefore, developing new antibiotics or therapeutic strategies is necessary to generate viable treatment options [13]. Numerous analogs to natural aminoglycosides have been designed to resist the action of resistance enzymes. The general strategy to achieve this purpose is to add or remove chemical groups to the natural molecule without affecting the antimicrobial properties, i.e., the capabilities to interact with the ribosome and produce a deleterious effect on the health of the bacterial cell. At the same time, the new molecule must become refractory to the action of aminoglycoside-modifying enzymes [14,15,16]. These compounds are known as semisynthetic aminoglycosides and have been successfully introduced into the clinical setting to treat resistant infections [17,18]. The first semisynthetic aminoglycoside, dibekacin (3′,4′-dideoxy-kanamycin B), was introduced in 1975 (Figure 1) [19]. The rationale for designing this aminoglycoside was that removing hydroxyl groups that are targets of *O*-phosphorylation would result in a molecule that is no longer a substrate of some aminoglycoside phosphotransferases [19]. Later, other semisynthetic aminoglycosides were synthesized with substituents that made them refractory to enzymatic modification or located at positions where the resistance enzymes introduce the chemical group to inactivate them. Others include combinations of removal of the target groups plus the addition of chemical groups at other locations. A few examples of these antimicrobials are amikacin, netilmicin, arbekacin, and isepamicin (Figure 1) [20,21,22,23]. In particular, amikacin has been widely used to treat multiresistant infections with great success [17]. Unfortunately, enzymes capable of catalyzing its inactivation started to appear and disseminate [10,24]. One of them, the aminoglycoside 6′-*N*-acetyltransferase type Ib [AAC(6′)-Ib], coded for by a gene that is present in integrons, transposons, and plasmids, spread throughout the world and became prevalent in most Gram-negative bacteria [12,25]. It is, therefore, imperative that research efforts to overcome resistance continue unabated.

Plazomicin is a next-generation semisynthetic aminoglycoside designed by modifying sisomicin by the addition of a 2(S)-hydroxy aminobutyryl group at the N1 position and a hydroxyethyl substituent at the 6′ position (Figure 1) [26,27]. These modifications make a molecular structure refractory to most aminoglycoside-modifying enzymes [10,28,29,30,31]. Plazomicin was approved in 2018 by the FDA to be used in patients with limited or no options for alternative treatment. It is active against multidrug-resistant Enterobacterales, including strains producing carbapenemases and extended-spectrum β-lactamases, while showing tolerable levels of nephrotoxicity and ototoxicity [29,32,33]. Unfortunately, despite the substitutions that make plazomicin a non-substrate for most aminoglycoside-modifying enzymes, the AAC(2′)-Ia enzyme identified in the chromosome of *Providencia stuartii* can catalyze the inactivation of the antibiotic molecule through transferring an acetyl group from the donor substrate acetyl-CoA to the amino group at the C-2′ position [34,35]. The crystal structures of this enzyme in complex with plazomicin and three other semisynthetic aminoglycosides have been recently reported [35,36]. Although AAC(2′)-Ia is not usually found in clinical isolates, it is a matter of time before it disseminates and becomes prevalent if the use of plazomicin increases. An obvious path to deal with the rise and dissemination of aminoglycoside-modifying enzymes is the continuous design of semisynthetic aminoglycosides. However, designing new generations of semisynthetic aminoglycosides has proven costly and time-consuming. These stumbling blocks warrant exploring alternative strategies, such as the developing inhibitors of the enzymatic inactivation that, together with the aminoglycoside, form a combination therapy effective against resistant pathogens [13]. The recent finding that metal ions, some of them complexed to ionophores (compounds that facilitate the internalization of ions inside the cell), inhibit the acetylation of aminoglycosides catalyzed by enzymes such as AAC(6′)-Ib, AAC(6′)-Ie, AAC(2′)-Ic, AAC(3)-Ia, AAC(3)-Ib, AAC(3)-IV, and Eis, and induce a reduction in the minimal inhibitory concentration (MIC) of several aminoglycosides to susceptibility levels proved the feasibility of this concept. The experimental results increased expectations that multidrug-resistant infections could be treated by these combination therapies [37,38,39,40,41,42,43]. In particular, Ag^+^ inhibits the inactivation by acetylation of amikacin elicited by AAC(6′)-Ib and reverses resistance in bacteria in culture at low concentrations without needing an ionophore [42]. This article describes the inhibition of AAC(2′)-Ia-mediated plazomicin-resistance by Ag^+^ in *Escherichia coli* harboring a recombinant clone containing the *aac(2′)-Ia* gene.

## 2. Results

### 2.1. Effect of Ag^+^ on AAC(2′)-Ia-Catalyzed Acetylation of Plazomicin

**Figure 2:**
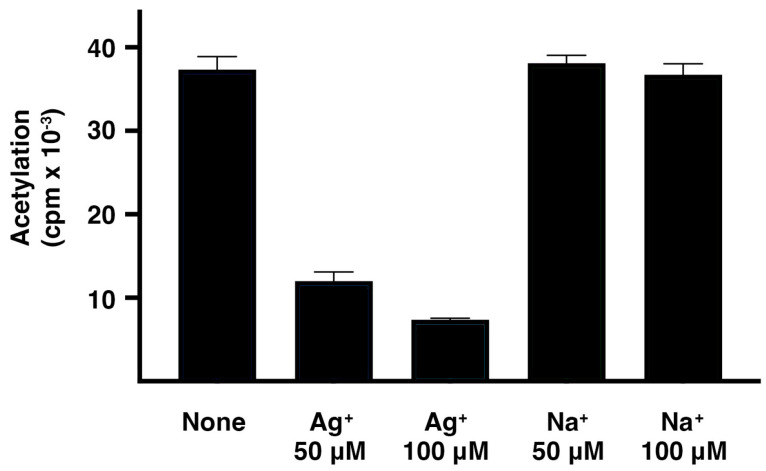
Effect of Ag^+^ on AAC(2′)-Ia activity. Assays were performed in triplicate by the phosphocellulose paper binding method with soluble extracts obtained from *E. coli* TOP10(pUC57AAC2Ia) cells. The reaction mixture contained 200 mM Tris HCl pH 7.6 buffer, 0.25 mM MgCl_2_, 330 μM plazomicin, the indicated concentrations of silver acetate (Ag^+^) or sodium acetate (Na^+^), and 0.05 μCi of [acetyl-1-^14^C]-acetyl-coenzyme A (specific activity 60 μCi/μmol) in a final volume of 30 μL. The enzymatic reactions were allowed to proceed at 37 °C. After 30 min incubation, 20 μL were spotted on phosphocellulose paper strips, which were immersed in water at 80 °C. Following this, the paper strips were washed twice by immersion in room temperature water. Finally, the phosphocellulose paper strips were left to dry and counted to determine the bound radioactivity, which corresponds to acetylated plazomicin.

Ag^+^ ions drastically interfered with the acetylation of plazomicin (Figure 2). The presence of silver acetate in the reaction mixture produced significant inhibition. These results, taken together with previous research showing that metal ions can inhibit the enzymatic acetylation of aminoglycosides, are an encouraging indication that Ag^+^ could serve as an adjuvant to plazomicin if AAC(2′)-Ia or a similar enzyme disseminates among bacterial pathogens. Control assays carried out in the presence of sodium acetate showed the same levels of acetylation as those in reactions with no additions (Figure 2). 

### 2.2. Effect of Ag^+^ on AAC(2′)-Ia-Mediated Resistance to Plazomicin

As determined using commercial E-strips, the *E. coli* TOP10(pUC57AAC2Ia) MIC of plazomicin was 12 μg/mL. To assess the effect of Ag^+^ on the resistance to plazomicin of growing cells, *E. coli* TOP10(pUC57AAC2Ia) was cultured in the presence of silver acetate in addition to plazomicin. A growth-level reduction was observed when only plazomicin was present (Table 1). However, the cells grew to an OD_600_ consistent with heavy growth, confirming that AAC(2′)-Ia confers substantial resistance to the antibiotic. Addition of silver acetate at 4 μM was sufficient to completely inhibit growth in the presence of plazomicin at a sub-MIC concentration (4 μg/mL) (Table 1). Furthermore, when the concentration of plazomicin was 8 μg/mL, still a sub-MIC value, 2 μM silver acetate was enough to inhibit growth (Table 1). A control experiment adding sodium acetate showed the same growth levels in the absence or presence of the addition (Table 1). The results described in this section unequivocally indicated that Ag^+^ interferes with resistance to amikacin mediated by AAC(6′)-Ib.

Studies on the expression of the *aac(2′)-Ia* gene suggest that in its natural location, the *P. stuartii* chromosome, is subjected to regulation [46]. To discard any regulatory role in the action of Ag^+^, the AAC(2′)-Ia open reading frame was placed downstream of the *bla*_TEM-1_ promoter and cloned using pUC57 as cloning vector to generate the recombinant plasmid pUC57PBLAAAC2Ia. The MIC of plazomicin of *E. coli* TOP10(pUC57PBLAAAC2Ia) was 12/16 μg/mL. Table 2 shows that the results of this experiment were similar to those observed with the gene under the control of the natural promoter. The resistance levels in liquid medium were slightly lower with the gene that carries the *bla*_TEM-1_ promoter. However, inhibition of resistance by Ag^+^ was identical with both promoters.

### 2.3. Bactericidal Effect 

**Figure 3:**
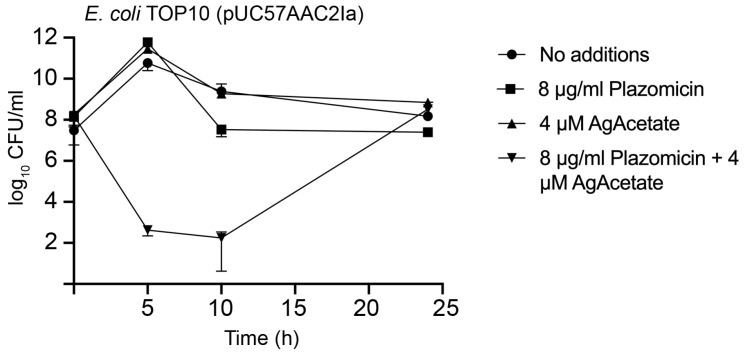
Time-kill assays for plazomicin in the presence of silver acetate. *E. coli* TOP10(pUC57AAC2Ia) was incubated at 37 °C until the cell concentration reached the CFU/mL indicated in the figure at time zero. At this moment, the culture was divided in four aliquots. An aliquot was not supplemented with any reagent. The others were each supplemented with 8 μg/mL plazomicin, 8 μM silver acetate, or both. The cultures were then incubated at 37 °C and the CFU/mL values were determined after the indicated periods. AgAcetate, silver acetate. Assays were done in duplicate, and the values are mean ± SD of two independent experiments.

Plazomicin showed bactericidal activity in previous studies [47]. Time-kill assays were used to evaluate if the phenotypic conversion to susceptibility observed when *E. coli* TOP10(pUC57AAC2Ia) was cultured in the presence of silver acetate in addition to plazomicin was due to a bactericidal effect. Figure 3 shows that adding silver acetate and plazomicin at a sub-MIC concentration had a robust bactericidal effect. Conversely, healthy growth was observed when one of the components was omitted. As in a previous report [47], regrowth was observed after 10 h incubation. It is worth noting that in the past study, regrowth in time-kill assays was observed with concentrations of up to 2× or 4× MIC values depending on the strain assayed. The bases for regrowth remain to be elucidated. Possible causes are the emergence of resistance or the presence of tolerant variants in the culture. The results of the experiments described in this section demonstrated that plazomicin, in the presence of Ag^+^ ions, exerted bactericidal action on *E. coli* cells, in which resistance is caused by the presence of the AAC(2′)-Ia enzyme. The bactericidal effect of plazomicin plus Ag^+^ ions on *E. coli* resistant cells was similar to that of plazomicin alone on susceptible *E. coli* cells.

### 2.4. Cytotoxicity of the Mix Plazomicin/Silver Acetate

**Figure 4:**
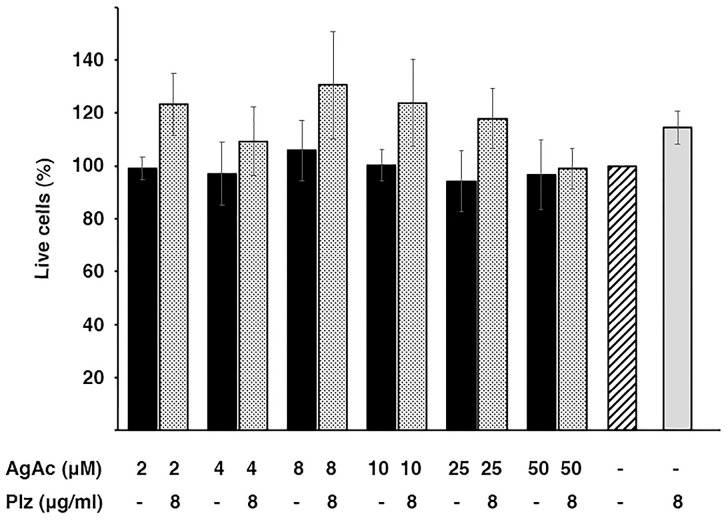
Cytotoxicity of silver acetate and plazomacin. Cytotoxicity on HEK293 cells was assayed using a LIVE/DEAD kit as described in the Materials and Methods section. The percentage of surviving cells was calculated relative to cells untreated (striped bar). Control of maximum toxicity was determined by incubating the cells in 70% methanol. Assays were carried out in triplicate and the values are mean ± SD of three independent experiments.

An essential factor for the viability of combination therapies is that they show low toxicity to the host. A preliminary analysis of the cytotoxicity of the mix investigated in this work was carried out using HEK293 cells. Figure 4 shows that the exposure of the cells to the combination or the individual components at the active concentrations did not cause significant mortality. While these experiments are a preliminary step toward understanding the toxicity of the combination plazomicin/silver acetate, the results warrant further development towards overcoming the action of the AAC(2′)-Ia enzyme.

## 3. Discussion

Bacterial infectious diseases are a major cause of premature death, compromised health, and sometimes disability caused by permanent sequelae [48,49,50]. Outbreaks of bacterial infection, usually associated with multidrug resistance, are increasingly reported and may soon be responsible for millions of deaths per year [51,52,53]. Furthermore, the increase in hard-to-treat or untreatable bacteria also threatens medical procedures such as surgery, care for premature infants, organ transplants, treatment of numerous chronic diseases, including cancer, and also multiple dental procedures [13,54,55,56]. The impact of the drug resistance crisis is such that it was included within the group of the top ten global health threats [57]. Compounding the problem, unlike in the past when new antibiotics were available if existing ones became ineffective, the number of new antibiotics in development is dangerously low [1]. It is necessary to devise methodologies that extend the life of antibiotics currently in use. The development of inhibitors of mechanisms of resistance that can be administered in combination with the cognate antibiotic can be a viable strategy to treat resistant bacteria [11,13]. Although there are no inhibitors of resistance to aminoglycosides in clinical use, this course of action has already been proven successful for β-lactamase-mediated resistance to β-lactams [58]. 

While plazomicin is a new aminoglycoside antibiotic, there are already enzymes that can inactivate it. AAC(2′)-Ia catalyzes the inactivation of plazomicin by acetylation and reduces its potency [28]. Following the steps of previous work indicating that selected metal ions interfere with the acetylation reaction [37,38,39,40,42,43,59], we tested the effect of Ag^+^. We chose this ion because, unlike other metals, previous work showed that ionophores were not necessary to observe reversion of resistance in growing bacterial cells when tested as a potential inhibitor of aminoglycoside resistance mediated by AAC(6′)-Ib [42]. Plazomicin was readily acetylated in vitro in a soluble extract of cells containing a recombinant clone harboring *aac(2′)-Ia.* The AAC(2′)-Ia activity results in resistance to plazomicin as determined by measuring MIC values for *E. coli* cells carrying recombinant clones that include the gene expressed under the control of the natural or the constitutive *bla*_TEM_ promoters. Since both recombinant plasmids were generated using the same plasmid vector, the gene dosage in both strains must be identical. Therefore, the similarity of the MIC values showed by both strains suggests that the described transcriptional regulation of expression of *aac(2′)-Ia* [60] does not impact resistance levels in the conditions used in our assays. The effect of silver acetate was also identical in the strains harboring the structural gene expressed under the control of both promoters. When plazomicin was present at 4 or 8 μg/mL, the concentrations needed to overcome resistance were as low as 1 and 2 μM, respectively. It is worth noting that it has been described that silver ions potentiate the effect of aminoglycoside and other antibiotics by mechanisms still in discussion. There is consensus that silver ions increase membrane permeability, which can enhance the effect of antibiotics against Gram-negative bacteria [61,62,63]. It has also been suggested that silver-mediated increased production of reactive oxygen species is one of the causes of potentiation of the aminoglycoside antibacterial effect [62]. However, this latter possibility has not been confirmed. Our prior [42] and present results indicate that silver ions also enhance the action of aminoglycosides by interfering with enzymatic inactivation, at least in the case of two aminoglycoside-modifying enzymes (AAC(6′)-Ib and AAC(2′)-Ia). We conclude that silver ions potentiate aminoglycosides by multiple mechanisms that result in the observed phenotypic conversion to susceptibility. These facts, taken together with the confirmation that plazomicin retains its bactericidal action when acting in concert with Ag^+1^ to inhibit the growth of resistant bacteria and that the combination plazomicin/silver acetate at the active concentrations does not exhibit cytotoxicity, make these mixes excellent candidates to extend the useful life of plazomicin and other aminoglycosides.

## 4. Materials and Methods

### 4.1. Bacterial Strains and Plasmids

*Escherichia coli* TOP10 F^-^ *mcrA* Δ(*mrr-hsd*RMS-*mcr*BC) Φ80*lac*ZΔM15 Δ*lac*X74 *rec*A1 *ara*D139 Δ(*ara-leu*)7697 *gal*U *gal*K *rps*L(Str^R^) *end*A1 *nup*G was transformed with the plasmid pUC57AAC2Ia and used for all assays. The plasmid pUC57AAC2Ia was constructed inserting the *P. stuartii aac(2′)-Ia* gene (accession number L06156, nucleotides 12-820) [60] into the *Bam*HI/*Hin*dIII sites of pUC57. The plasmid pUC57PBLAAAC2Ia was generated fusing the *bla*_TEM_ promoter and Shine-Dalgarno sequences, fragment encompassing nucleotides 4154-4225 (reverse complement, accession number J01749) [64] to the *aac(2′)-Ia* open reading frame (fragment encompassed by nucleotides 264-810, accession number L06156) [60]. Transformation of *E. coli* TOP10 with pUC57AAC2Ia or pUC57PBLAAAC2Ia was performed as recommended by the supplier of the competent cells (Invitrogen, Waltham, MA, USA).

### 4.2. Bacterial Growth

Bacteria were cultured in Lennox L broth (1% tryptone, 0.5% yeast extract, 0.5% NaCl) with the addition of 2% agar in the case of solid medium. Plazomicin resistance levels were determined in cation-adjusted Mueller-Hinton broth. Culturing was carried out at 37 °C in a shaker. Growth was assessed determining the optical density at 600 nm (OD_600_) of the cultures containing the specified additions. Silver ions were added as silver acetate due to its adequate solubility in water. Plazomicin was generously supplied by Cipla Therapeutics (Mumbai, India).

### 4.3. MIC Determination 

MIC values were measured using plazomicin commercial E-strips (Liofilchem S.r.l., Roseto degli Abruzzi, Italy) following the recommendations of the supplier on Mueller-Hinton agar plates. The strips were applied to the Muelle-Hinton agar plates, which were incubated overnight at 37 °C. The next morning, values were determined at the intersection of the strip MIC reading scale and the growth ellipse.

### 4.4. Time-Kill Assays 

Bacterial cells were cultured in cation-adjusted Mueller-Hinton broth until they reached 10^7^ CFU/mL. At this time the cultures were divided in four aliquots, one of them was left intact and the others were supplemented with either 8 μg/mL plazomicin, 4 μM silver acetate, or both. Incubation was continued at 37 °C with shaking and the CFUs were determined after the indicated times [40]. 

### 4.5. Acetyltransferase Assays 

Total soluble proteins (enzymatic extracts) were prepared as before [42]. Briefly, cells were pelleted from cultures by centrifugation and resuspended in a 0.5 mM MgCl_2_ solution. The cells were lysed by sonication with a Heat Systems Ultra-sonic, Inc., Model No. H-IA (Plainview, NY, USA) cell disrupter. The soluble protein fraction was then separated from unbroken cells, membranes, and cell debris by centrifugation in a microfuge for 10 min at 4 °C. The protein concentration of the extracts was measured using a commercial reagent (Bio-Rad Protein Assay). Acetyltransferase activity was assessed using the phosphocellulose paper binding assay [65]. Soluble extract (120 μg protein) obtained from *E. coli* TOP10(pUC57AAC2Ia) cells was added to the reaction mixture (200 mM Tris HCl pH 7.6 buffer, 0.25 mM MgCl_2_, 330 μM plazomicin, the indicated concentrations of sodium acetate or silver acetate, and 0.05 μCi of [acetyl-1-^14^C]-acetyl-coenzyme A (specific activity 60 μCi/μmol). The reaction mixture final volume was 30 μL. Silver ions were added as silver acetate due to its adequate solubility in water. After incubating the reaction mixture at 37 °C for 30 min, 20 μL were spotted on phosphocellulose paper strips. The unreacted radioactive substrate [acetyl-1-^14^C]-acetyl-coenzyme A was removed from the phosphocellulose paper strips by submersion in 80 °C water followed by two washes by submersion in room temperature water. After this treatment, the only radioactive compound bound to the phosphocellulose paper strips was the acetylated plazomicin. The phosphocellulose paper strips were then dried and the radioactivity corresponding to enzymatic reaction product was determined in a scintillation counter.

### 4.6. Cytotoxicity Assays

Cytotoxicity was assessed on HEK293 cells [66]. The methodology followed was described previously [67]. Cytotoxicity was determined for of the combination silver acetate/plazomicin at various concentrations. One thousand cells per well were cultured on flat-bottom 96-well, black microtiter plates. After 12 h incubation, the testing compounds were added at the desired concentrations and incubation was continued for 24 h. Following, the cells were washed with sterile D-PBS, resuspended in the LIVE/DEAD reagent (2 μM ethidium homodimer 1 and 1 μM calcein-AM) (Molecular Probes), and incubated for 30 min at 37 °C. At this moment the fluorescence levels corresponding to dead and live cells (645 nm and 530 nm, respectively) were measured. The percentage of dead cells was calculated relative to the untreated cells. Maximum toxicity was calculated treating cells with 70% methanol for 20 min. Experiments were conducted in triplicate. The results were expressed as mean ± SD of three independent experiments. HEK293 cells were purchased from BEI resources (Manassas, VA, USA), catalog number NR-9313.

## 5. Conclusions

Antibiotic resistance could become the next pandemic. There is an urgent need for novel strategies to extend the useful life of antibiotics currently in use. The Centers for Disease Control Antibiotic Resistance Threats in the United States Report of 2019 called attention to the magnitude of the problem. They warned that relying only on new antibiotics would be unwise to deal with the crisis [68]. Latest-generation antibiotics such as plazomicin can quickly be overcome by resistance mechanisms developed by bacteria. The ability of Ag^+^ to interfere with the action of AAC(2′)-Ia, an aminoglycoside-modifying enzyme that mediates the inactivation of plazomicin, makes it an excellent candidate as a plazomicin adjuvant to eliminate this enzyme as a threat to the effectivity to this antibiotic. The low cytotoxicity observed at the active concentrations makes the combination plazomicin/Ag^+^ a viable option for treating multidrug-resistant infections.

## Figures and Tables

**Table 1:** Growth in the presence of plazomicin and silver acetate.

Plazomicin (μg/mL)	Silver Acetate (μM) OD_600_				Sodium Acetate (μM) OD_600_	
	0	1	2	4	0	8
0	3.21 ± 0.02	3.09 ± 0.01	3.09 ± 0.02	3.18 ± 0.11	3.04 ± 0.06	3.12 ± 0.08
4	3.05 ± 0.08	2.86 ± 0.11	1.95 ± 0.01	0.12 ± 0.01	1.39 ± 0.03	1.34 ± 0.06
8	1.21 ± 0.01	1.26 ± 0.01	0.10 ± 0.03	0.02 ± 0	1.13 ± 0.02	1.08 ± 0.04

Cultures of *E. coli* TOP10(pUC57AAC2Ia) were performed in cation-adjusted Mueller Hinton without additions or supplemented with silver acetate or sodium acetate at the indicated concentrations.

**Table 2:** Growth in the presence of plazomicin and silver acetate.

Plazomicin (μg/mL)	Silver Acetate (μM) OD_600_			
	0	1	2	4
0	3.54 ± 0.01	3.48 ± 0.02	3.50 ± 0.07	3.35 ± 0.10
4	1.71 ± 0.02	1.66 ± 0.09	0.95 ± 0.09	0.09 ± 0.07
8	0.68 ± 0.06	0.24 ± 0.02	0.14 ± 0.06	0.03 ± 0

Cultures of *E. coli* TOP10(pUC57PBLAAAC2Ia) were performed in cation-adjusted Mueller Hinton without additions or supplemented with silver acetate or sodium acetate at the indicated concentrations.

## Data Availability

Data is contained within the article.
